# Educational application for visualization and analysis of electric field strength in multiple electrode electroporation

**DOI:** 10.1186/1472-6920-12-102

**Published:** 2012-10-30

**Authors:** Samo Mahnič-Kalamiza, Tadej Kotnik, Damijan Miklavčič

**Affiliations:** 1 Faculty of Electrical Engineering, University of Ljubljana, Tr&2;aška 25, Ljubljana, SI-1000, Slovenia

**Keywords:** Education on electroporation, Electromagnetic field visualization, Applications in subject areas, Interdisciplinary projects, Interactive learning environments

## Abstract

**Background:**

Electrochemotherapy is a local treatment that utilizes electric pulses in order to achieve local increase in cytotoxicity of some anticancer drugs. The success of this treatment is highly dependent on parameters such as tissue electrical properties, applied voltages and spatial relations in placement of electrodes that are used to establish a cell-permeabilizing electric field in target tissue. Non-thermal irreversible electroporation techniques for ablation of tissue depend similarly on these parameters. In the treatment planning stage, if oversimplified approximations for evaluation of electric field are used, such as U/d (voltage-to-distance ratio), sufficient field strength may not be reached within the entire target (tumor) area, potentially resulting in treatment failure.

**Results:**

In order to provide an aid in education of medical personnel performing electrochemotherapy and non-thermal irreversible electroporation for tissue ablation, assist in visualizing the electric field in needle electrode electroporation and the effects of changes in electrode placement, an application has been developed both as a desktop- and a web-based solution. It enables users to position up to twelve electrodes in a plane of adjustable dimensions representing a two-dimensional slice of tissue. By means of manipulation of electrode placement, i.e. repositioning, and the changes in electrical parameters, the users interact with the system and observe the resulting electrical field strength established by the inserted electrodes in real time. The field strength is calculated and visualized online and instantaneously reflects the desired changes, dramatically improving the user friendliness and educational value, especially compared to approaches utilizing general-purpose numerical modeling software, such as finite element modeling packages.

**Conclusion:**

In this paper we outline the need and offer a solution in medical education in the field of electroporation-based treatments, e.g. primarily electrochemotherapy and non-thermal irreversible tissue ablation. We present the background, the means of implementation and the fully functional application, which is the first of its kind. While the initial feedback from students that have evaluated this application as part of an e-learning course is positive, a formal study is planned to thoroughly evaluate the current version and identify possible future improvements and modifications.

## Background

### Electroporation

Electroporation is a term coined in the early 1980's [1] for phenomena already described more than a decade earlier [2], when an interesting property of biological membranes has been observed. Electroporation mainly results in transient increase in membrane permeability when these membranes – which are mainly composed of lipid bilayers – are exposed to very short and intense electric fields. The physical mechanism responsible for increased permeability is thought to be the formation of nano-scale defects termed *pores* (thus electro*poration*) in the lipid structure [3,4]. This allows molecules that usually do not cross the membrane to cross it with relative ease, though the exact mechanism of this transport remains a subject of scientific debate [5]. In case the electric field strength is too high, changes to membrane structures and consequently to the cell are not temporary, but instead result in cell death, a phenomenon known as irreversible electroporation. On the other hand, if the cells survive, increased permeability of membrane is only temporary and the phenomenon is termed reversible electroporation. This observed transient increase in permeability of electroporated membranes of biological cells has offered opportunities for extensive research and development resulting in a number of applications of electroporation, such as gene (DNA) delivery, introduction of drugs into cells, fusion of cells, electrochemotherapy for treatment of cancer, gene delivery in tissue and transdermal delivery of drugs and genes as well as a number of other applications, see for instance [6,7] for an overview. In these applications viability of the porated cells is important and thus irreversible electroporation is consciously avoided and generally considered to be an undesired side effect. However, there are also interesting medical applications of irreversible electroporation [8], such as non-thermal irreversible electroporation for tissue ablation. Irreversible electroporation is also rapidly entering the domains of industrial [9,10] and environmental [11] applications.

### Electrochemotherapy

Electrochemotherapy (ECT) is a local treatment that successfully combines application of cell-membrane-permeabilizing electric pulses and chemotherapeutic drugs. For successful ECT, the pulses must be of adequate amplitude, duration, number, repetition frequency and shape [12], in order to achieve local increase in uptake and hence cytotoxicity of otherwise non-permeant or poorly permeant anticancer drugs. Two drugs have been identified as candidates for ECT: bleomycin and cisplatin [13,14]. The transport of bleomycin across the nonpermeabilized plasma membrane is highly limited [15]. Using electroporation to increase membrane permeability provides bleomycin with access to cytosol and DNA, where it causes DNA breakdown. The cytotoxicity of bleomycin is thereby increased by a factor of several thousand [16]. In short, the main mechanism of ECT is the electroporation of cells in tumors and consequent increase in drug effectiveness by enabling the drug to reach intracellular targets. For other supporting mechanisms, see [17,18].

Since all clonogenic cells in the tumor need to be eradicated for effective treatment, all cells have to be permeabilized, i.e. all cells in the tumor have to be exposed to appropriate electric pulses. This means that the effectiveness of electrochemotherapy depends on both the drug availability in the tumor and coverage of the whole target area by a sufficiently high electric field [19].

There are several factors in ECT of tumors that we must consider before we can confidently assume we have successfully electroporated the entire tumor area. First, there is the issue of tumor and surrounding tissue electrical properties that may exhibit considerable inhomogeneity. Conductivity is of particularly high importance for example, since higher tumor conductivity compared to surrounding tissue results in lower electric field strength in the tumor and undesirably high fields in the surrounding healthy area that may cause irreversible electroporation [20]. Second, there is the issue of electrodes used to apply the pulses, mainly of their shape, size and position. In general, there are two types of electrodes – plate and needle electrodes. Plate electrodes are non-invasive, usually parallel and with either a fixed or adjustable distance in between the two plates, whereas needle electrodes are used invasively, which ensures good electrical contact, but has other drawbacks. In using plate electrodes, we must ensure good electrical contact and optimal distance between electrodes to fit the tumor [21,22] and with needle electrodes – since electric field distribution is more inhomogeneous and dependent on their diameter, distance between them and depth of insertion [23] – the task of ensuring entire tumor area coverage is even more arduous [19].

Several papers have been published based on numerical modeling, e.g. [19,24] and in vivo studies [25] on importance of tumor coverage by sufficiently high electric fields for successful ECT, demonstrating the need for careful consideration and control of variables. Effectiveness of ECT has been confirmed in a study conducted by a consortium of four medical institutions gathered in the ESOPE (European Standard Operating Procedures of Electrochemotherapy) project, in the scope of which standard operating procedures for ECT were published [26]. However, in advanced cases such as intracranial [27] or liver metastasis tumor treatment [28], the need for patient-specific treatment planning has been highlighted and is being developed [29,30].

### Non-thermal irreversible electroporation for tissue ablation and other techniques

Irreversible electroporation has been studied extensively in recent years as a method of tissue ablation. While the high number of applied pulses used to achieve classical irreversible electroporation may also cause thermal damages due to Joule heating of the current-conducting tissue, non-thermal irreversible electroporation (NTIRE) is of particular interest since it results in well-defined areas of tissue ablation and no protein coagulation [31]. The potential clinical applications of NTIRE are therefore numerous. It is technically a simple procedure, requiring only the insertion of electrode needles without any addition of chemotherapeutic as with ECT; it is fast in comparison to other ablation methods such as RF ablation or cryoablation; can be monitored with ultrasound; produces a sharp delineation between treated and untreated regions; and affects only cells while sparing the connective structure [8,32]. Since it has been observed that NTIRE spares the vascular structure and nerves, there is hope of using this technique in treatment of tumors abutting large blood vessels [33]. Promising as this treatment is, we are faced with similar considerations as with reversible electroporation in the case of ECT or its many other applications, namely to ensure adequate local electric field strength, sufficient for irreversible, yet non-thermal electroporation.

In addition to NTIRE and ECT, a number of other minimally invasive therapies for treatment of deep-seated tumors (particularly those of the liver) exist, e.g. high intensity focused ultrasound, microwave ablation and radiofrequency ablation. The latter is a minimally invasive procedure where a needle electrode is inserted percutaneously into a tumor, much as in ECT or NTIRE. The electrode acts as an antenna for electromagnetic waves emitted by a generator; the tumor is heated up and thereby destroyed [34]. As with NTIRE or ECT, the need for accurate needle placement exists and its importance has been evaluated. Success, i.e. improved treatment, has been proven to be correlated to the experience of the operator [34].

### The need for education on electroporation

**Figure 1:**
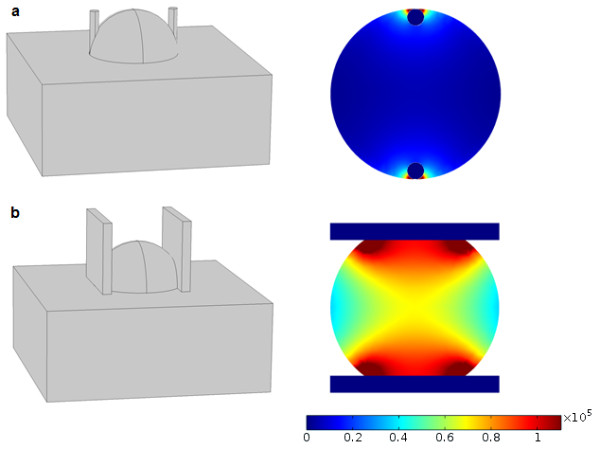
**Electric field distribution for plate electrodes in a cutaneous tumor electroporation (a) and parallel needle electrodes in cutaneous tumor electroporation (b).** Geometry is shown on the left hand side and resulting field strength on the right. Tumor diameter in both instances is 2 cm, electrode thickness 0.2 cm and electrode distance 1.6 cm. The applied voltage was 1300 V. Resulting field strength on the color scale is in volts per meter.

As outlined in the paragraphs above, both reversible and irreversible electroporation are promising approaches in treating solid tumors, however their success is highly dependent on local field strength. Poorly defined or unknown properties such as tissue conductivities and inhomogeneities in addition to our choice of electrode type, positions and pulse amplitudes, affect the electric field distribution. With many factors to take into consideration, we may dismiss important uncontrolled variables or use a well-studied but irrelevant model that cannot be applied to a specific situation. An example of this is the persistent use of a very simple approximation for electric field strength that holds only under very particular conditions. This frequently used approximation equates the electric field strength *E* to the voltage *U* applied on the electrodes divided by the distance *d* between the electrodes, i.e. *E* ≈ *U*/*d*. Since the local electric field strength has been identified as the key factor in achieving both reversible and irreversible electroporation (see [19] for details), i.e. local *E* has to reach a certain threshold value *E*_rev_ or *E*_irrev_, it is the parameter we most often optimize our electrode configuration for. There is a problem, however, since this simple relation is strictly valid only in an isotropic homogeneous medium between two infinitely large parallel plate electrodes. When using parallel plate electrodes in ECT or NTIRE, this simple model best approximates the actual field strength far from the electrode edges, provided the electrode width is sufficiently large compared to the electrode distance. In any case, the approximate model fails completely as we approach the edges (see Figure 1a). Results are even worse in the case of needle electrodes, where of course there are no parallel plates to speak of (see Figure 1b and [35] for further elucidation). Therefore, using the simple parallel plate approximation for *E* in individual cases of tumor treatment could possibly result in significant reduction of response in treatment for which the complete response rate has been shown to be as high as 74% [17].

As ECT and NTIRE are steadily finding their way into more and more clinics worldwide [36-39] and establishing themselves as safe and effective treatments of cancer, it is becoming more and more important that medical personnel performing and planning ECT (or NTIRE) is aware of their mechanisms of action, the underlying physics and possible pitfalls. This paper presents an application for visualization and analysis of the electric field strength in multiple-needle-electrode electroporation aimed at users with basic knowledge of the principles of electroporation, but lack of education in physics and engineering who may therefore need visual, interactive and didactic instruments to gain a deeper understanding of the principles necessary for effective use of these promising treatments. Since educational applications of the sort are scarce and research is focused primarily on advancing the field rather than educating, the need to explore options and applications in this area of engineering education seems pressing and applicative results should be welcome. The application is part of a now firmly established annual course Electroporation Based Technologies and Treatments, EBTT (http://www.ebtt.org). The e-learning laboratory exercise into which the presented application has been incorporated, has been developed and described previously [40].

## Implementation

### Theoretical background

The theoretical background for the mathematical engine that is at the heart of our application has been derived from the electrostatic field theory, more specifically, extended from a solution for electrostatic potential generated by two long parallel charged conductors given in [41]. We have omitted the detailed mathematical derivations here as they are outside the scope of this paper; however, we have included a derivation of the equations below in the Appendix for those readers that may be interested in further development or modifications to our model. Below, we specify only the final formulation of the mathematical model and the method of application of this analytical solution into the field-calculating engine that represents the core of Application for Visualization of Target Tissue in Electroporation (ApiVizTEP), since this is the only essential part of the application around which the user interface is built. Similarly to the approach that we present in this paper, other analytical models have been developed previously using complex analysis. For a six-needle array, one may find a first-order analytical approximation for the electric field strength in [42]. Other authors have expanded on this approach further, see e.g. [43] and [44]; however, these solutions do not give an accurate analytical expression for the electric field strength since quite possibly such a solution cannot be obtained for three or more electrodes active concurrently at a given time. Since currently electroporation devices mostly power only one pair of electrodes at a time [45], we have not pursued a solution with more than two concurrently active electrodes, thus avoiding the derivation of an approximate solution. Instead, we give an accurate solution for a two needle electrode system that is not based (mathematically) on previously published work.

The accurate analytical solution for electric field strength of two long parallel cylindrical conductors (needle electrodes) on a plane perpendicular to the conductor longitudinal axis (2-D solution), is given by the following equation

$$\left|E\left(x,y\right)\right|=C\cdot \sqrt{{\left(\frac{x{'}_{A}-x}{{\left(x{'}_{A}-x\right)}^{2}+{\left(y{'}_{A}-y\right)}^{2}}-\frac{x{'}_{B}-x}{{\left(x{'}_{B}-x\right)}^{2}+{\left(y{'}_{B}-y\right)}^{2}}\right)}^{2}+{\left(\frac{y{'}_{A}-y}{{\left(x{'}_{A}-x\right)}^{2}+{\left(y{'}_{A}-y\right)}^{2}}-\frac{y{'}_{B}-y}{{\left(x{'}_{B}-x\right)}^{2}+{\left(y{'}_{B}-y\right)}^{2}}\right)}^{2}}$$

$$\text{where}\;C=\frac{V_{\mathrm{AB}}}{2\cdot \log\frac{d_{\mathrm{AB}}+\sqrt{{d_{\mathrm{AB}}}^{2}-4\cdot {\rho_{0}}^{2}}}{2\cdot \rho_{0}}}$$

$$\begin{array}{ll} \text{and}\; & \begin{array}{ll} x{'}_{A}=x_{A}+\left|e\right|\cdot \cos\Theta_{1}, & \;\Theta_{1}=\arctan\frac{y_{B}-y_{A}}{x_{B}-x_{A}}\\ x{'}_{B}=x_{B}+\left|e\right|\cdot \cos\Theta_{2}, & \;\Theta_{2}=\arctan\frac{y_{A}-y_{B}}{x_{A}-x_{B}}\\ \begin{array}{l} y{'}_{A}=y_{A}+\left|e\right|\cdot \sin\Theta_{1},\\ y{'}_{B}=y_{B}+\left|e\right|\cdot \sin\Theta_{2} \end{array} & \;e=d_{\mathrm{AB}}/2-\sqrt{{\left(d_{\mathrm{AB}}/2\right)}^{2}-{\rho_{0}}^{2}} \end{array} \end{array}$$

*V*_AB_ is the voltage on an electrode pair (anode–cathode), *d*_AB_ is the distance between the electrodes, *ρ*_0_ is the electrode diameter, while *x*’_A_, *y*’_A_ and *x*’_B_, *y*’_B_ are coordinates of the center of the electrode mathematical equivalents (the electrical center *x*’_A_, *y*’_A_ and *x*’_B_, *y*’_B_ of the electrodes is eccentric to the actual geometrical center *x*_A_, *y*_A_ and *x*_B_, *y*_B_ due to finite i.e. non-zero electrode diameter).

Our application employs a mesh of 400 × 400 points and the electric field strength *E* is calculated for each of the 160.000 points using the expression above. The time needed to perform the necessary calculations and display the results is practically negligible, ranging from a few hundred milliseconds for two active electrodes up to one and a half seconds for twelve active electrodes using an average modern personal computer, i.e. at least a dual core 2 GHz processor equipped with 2 GB of memory or more. It needs to be emphasized that while the application allows for inclusion of up to twelve electrodes distributed into one to six groups with variable voltages, the application fundamentally breaks down these groups into anode–cathode pairs and calculates the electric field strength by means of the equations above for every pair. It then searches this set of results to obtain the maximum value of the electric field in each of the 160.000 points. This value represents the final solution in that point, since what we are interested in is determining the maximum value for the local field strength. In this way we have eliminated the need for a solution for the electric field of more than two concurrently active electrodes. Such an approach is justified by the fact that we have already mentioned – i.e. currently electroporation devices only power two electrodes at the same time.

### Tools and platforms

The application has been written in C# programming language in the Microsoft® Visual Studio 2010 integrated development environment. The library used was the Microsoft® .NET Framework version 4. The development environment and base library have both been selected based on personal preference and possibilities that are enabled by the enhancements in these tools concerning ease of graphical user interface (GUI) development by separating the markup that describes the GUI elements from programming logic that provides the functionality.

The internet version of the application is hosted on a semi-open platform, since a Microsoft® Windows operating system is hosting an Apache™ HTTP Server web server with installed PHP support and a free edition of Oracle® MySQL, providing a web interface to basically the same, though slightly modified and recompiled, engine as in the desktop version.

## Results

The application that we developed has been named Application for Visualization of Target Tissue in Electroporation, or ApiVizTEP in short. It is a Microsoft .NET application bundled into an installation package together with the .NET Framework version 4 Client Profile redistributable installation, which enables the user to install the application locally, provided the machine hardware and operating system support this prerequisite framework, as do most modern Windows operating systems installed on compatible hardware.

**Figure 2:**
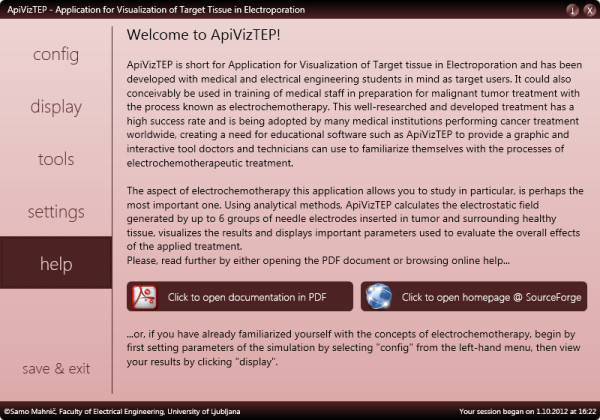
The help panel of ApiVizTEP with introduction and brief instructions.

At the start the application presents itself with a navigational panel on the left-hand side of the window and a help panel to the right (see Figure 2).

**Figure 3:**
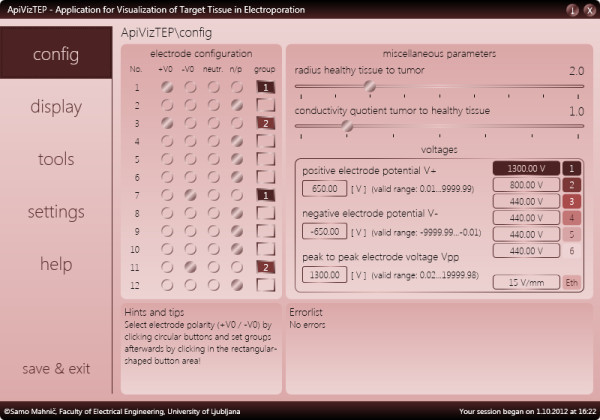
The configuration panel of ApiVizTEP with various options.

The help panel contains instructions on locating the configuration of the system, i.e. directs to the *config* and *settings* panels. These contain various model and system settings that we describe here briefly: It is possible to include from two up to twelve needle electrodes in the model that may be distributed into one, two, or more (up to six) groups. One cathode and one or more anodes or vice versa (one anode and one or more cathodes) form such a group. These are defined in electroporation studies in situations where applied voltages are different for different pairs (or groups in general) of electrodes, as currently electroporation devices mostly power only one pair of electrodes at the same time [45]. The applied voltages are configurable per group (see Figure 3). The reversible electroporation threshold *E*_rev_ as well as irreversible electroporation threshold *E*_irrev_ may also be set (default settings 300 V/cm and 800 V/cm, respectively), as these thresholds are highly dependent on the modeled tissue (e.g. bone vs. muscle vs. liver etc.) as well as on treatment parameters, e.g. number and duration of pulses [46]. These thresholds may be set in three different units: V/cm, V/mm, or V/in. There are also interface elements for changing the ratio of tumor size to healthy tissue area, and for modifying the conductivity ratio of tumor to healthy tissue, but these options are not, at present, taken into account in calculations of the field strength, although the effects of inhomogeneous conductivities can be significant (see Discussion, Evaluation of accuracy and usefulness of obtained solution).

**Figure 4:**
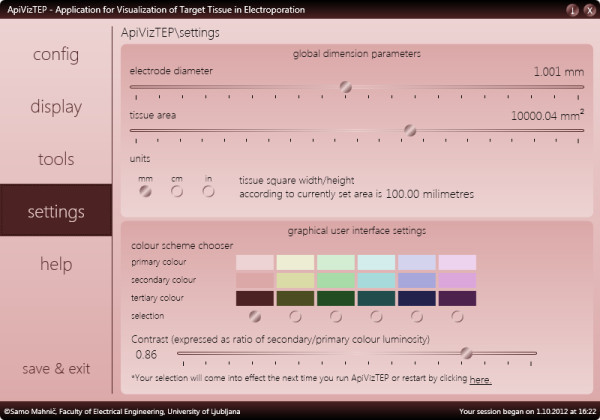
The settings panel of ApiVizTEP with various options.

An electroporation model studied in ApiVizTEP is flexible in terms of adjustable tissue area (1.61 mm^2^ to 16129.00 mm^2^) and electrode diameter (0.10 mm to 2.00 mm, see Figure 4). The user interface for configuring has also been designed with extensive error-checking capabilities to prevent users from misconfiguring the model.

**Figure 5:**
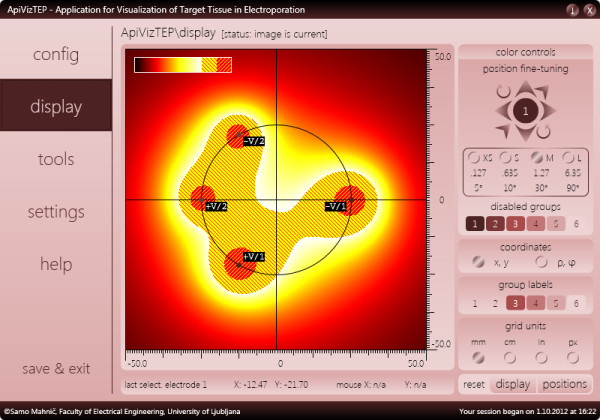
The display panel of ApiVizTEP with its 5 sub-sections.

**Figure 6:**
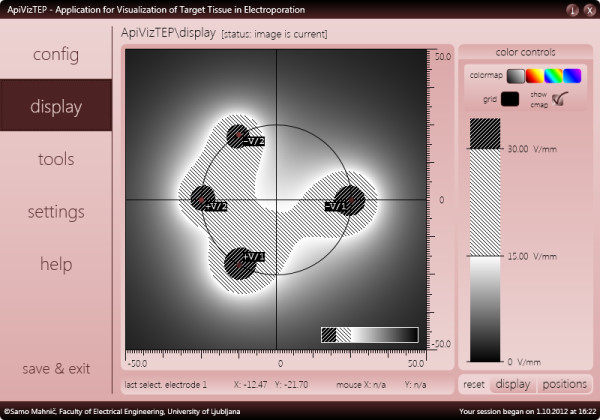
**The display panel of ApiVizTEP with the color controls sub-section of the display panel visible.** The color bar is annotated with important field strength values (thresholds) and a gray color scheme has been selected to illustrate the results display adaptability (see for comparison the same results displayed in “hot” color scheme in Figure 5).

Once satisfied with the configuration, the user may switch to the *display* panel, which contains the results part of the application (see Figure 5). This display panel is divided into five areas; (1) the tissue area with color-coded display of calculated electric field strength, electrode positions and miniature color bar (legend); (2) the large color bar and controls area (accessible via the *color controls* button, see Figure 6); (3) the position fine-tuning, coordinates and units panel; (4) the selected electrode position and mouse cursor position bar; and (5) the position and display settings reset bar. The tissue area, measuring 400 × 400 picture elements (pixels), is displaying the results of our model. There are also the X and Y axes and a circle representing the boundary between the tumor and the surrounding tissue. Currently, this boundary serves no particular function other than representing a target for our modeled ECT or NTIRE efforts, a point on which we will elaborate later. What we are seeing in this area is color-coded electric field strength, of which the values are encoded into color based on the chosen palette (color bar), shown in Figure 6.

As described in the Background, coverage of the entire target area with fields above reversible threshold is very important for successful ECT, and above irreversible threshold for ablation of tissue by NTIRE. Thus, the learning experience for the user is in the experimentation with the chosen setup, by one or more of the following methods of interaction: (1) repositioning of electrodes via mouse (drag and drop) or eight buttons by a defined increment (up, down, left, right, rotation around center point, towards or away from the center point); (2) adjusting the number of electrodes or the applied voltages (*configuration* panel); or (3) varying tissue and electrode dimensions (*settings* panel).

**Figure 7:**
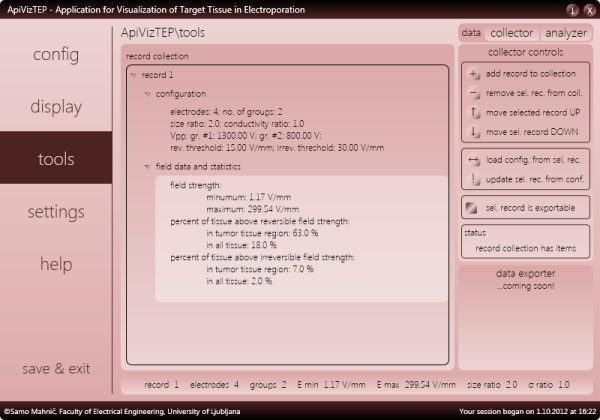
**The tools panel of ApiVizTEP with data collector showing analysis of results obtained with configuration and settings shown in Figures**3**and**4**, respectively.**

The *tools* panel of the application contains two useful analysis tools, one already developed (*data collector*) and the other currently under development (*data analyzer*). Data collector (as shown in Figure 7) is capable of maintaining a record of our selected configurations that we wish to analyze. An individual record holds information about the electrode and voltage settings as well as analysis performed at the instance of record creation; the maximum and minimum field strength, as well as percentage of tumor and surrounding tissue subjected to irreversible or reversible electric field strengths (see Figure 7).

ApiVizTEP also allows the user to save data via the *save & exit* panel. An option is available to either save configuration to a file that can be loaded at any time via a key combination, or automatically at the next startup of the application. Additionally, there is an option to retain the data collected in the data collector.

**Figure 8:**
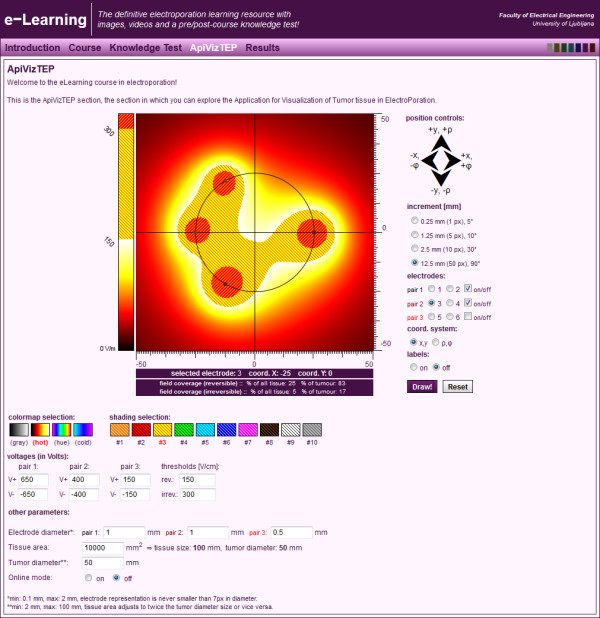
The web version of ApiVizTEP as part of an e-learning application on electroporation.

For platform cross-compatibility and accessibility, a web interface has been developed (Figure 8) that uses the same engine as the standalone application; in other words, the application has been recompiled without the graphical interface and the interface built as a web application. This is now part of an online e-learning course presented at the EBTT International Workshop [40].

## Discussion

The main result of the work presented in this paper is the educational application that calculates and provides visualization of the electric field in multiple needle electrode electroporation, such as is commonly used in electrochemotherapy [28] or tissue ablation by non-thermal irreversible electroporation [39]. It has been developed both as an installable desktop application and a web service with a graphical interface.

### ApiVizTEP from the user’s point of view

From the perspective of a potential user, the application has been designed in an attempt to be both easy to use and work with, and aesthetically pleasing. It is important for a modern educational piece of software to be not only functional in the educational sense, but also designed in a way that is inviting the user to “play” with the various features and modes of use, thus provoking a motivated self-paced learning experience [47]. We believe the design of our application has successfully incorporated these principles in the following sense; the amount of textual information related to the understanding of user interface functionality is minimal, the help section of the application is mainly concerned with bridging the gap in knowledge of the electroporation background and need not be focused on the interface as it is minimalistic in design; since we also aimed at flexibility in customizing and individualizing the interface by the users themselves, we offer several different color options and palettes for visualizing the data as well as six selectable color schemes of the graphical interface (see Figure 3 – graphical user interface settings); and finally, intelligent programming logic of error reporting and warnings prevents the user from misconfiguring or misusing the system and therefore failing to produce or obtain erroneous results. Thus far feedback from users, i.e. students at a workshop on electroporation, has been positive. While no formal study on the usability and user-friendliness has been performed, based on individual oral accounts of the learning experience, we can conclude that the application is easy to use and the graphical interface is intuitive; there have been no requests for clarification from users during courses that incorporated ApiVizTEP, while all participants successfully completed their assignments that were based on work with the application.

### Evaluation of accuracy and usefulness of obtained solution

With respect to the accuracy and reliability of the results in light of present knowledge on mechanisms of ECT and NTIRE, there are a couple of points to consider. First, it needs to be emphasized that due to optimization of speed with which we obtain our results within ApiVizTEP and our desire to enhance user experience – consequently using an analytical model – we are limited by the need for a low-complexity model of electroporation. The reason for this is that an analytical model may be described and solved without utilization of numerical methods which come with high computational costs. Of main concern in this regard are differences in electrical properties of tumor and surrounding tissue. Since tumors normally exhibit higher conductivity compared to the surrounding area [48], local electric field strength inside the tumor can be only a fraction of the field strength of the surrounding tissue [20]. This inhomogeneity in tissue conductivity is at the time of writing of this article incorporated into the graphical user interface of ApiVizTEP, i.e. the user may set tumor conductivity to surrounding tissue conductivity ratio, but our mathematical model and therefore the field-calculating engine do not take this ratio into account. We therefore treat the entire area as homogeneous in conductivity. The functionality of varying the tumor tissue to surrounding tissue conductivity ratio is built into the graphical user interface in anticipation of future work on developing the analytical model still further. If possible, the conductivity inhomogeneity will be accounted for in a future model, i.e. a future version of the application. If it is, however, impossible to obtain such a solution analytically, the application will be extended with a numerical engine.

Secondly, we would like to point out that our model is two-dimensional, as we have imagined our area of interest to be a slice of three-dimensional space. This simplification is made under the assumption that the generated field is homogeneous along the electrode insertion axis if we observe a plane that is distant from electrode end points. Since currently in ECT [28,29] and NTIRE [49-52] of deep-seated tumors the electrodes are normally inserted into tissue parallel to each other, the fact that we do not consider the third spatial dimension is not of particular concern.

## Conclusions

The research and development presented in this paper is grounded in decades of studying the phenomenon of electroporation and the development of electroporation-based technologies and treatments. It has resulted in a software product aimed at educating a relatively small but rapidly growing population of researchers and medical staff in fields where applications utilizing electroporation are growing in numbers, such as cancer treatment, food processing and the environment (e.g. waste water treatment, biofuel, etc.). As these applications mature into standard practices, a further increase in need for educational solutions of this kind will become a necessity, especially in the field of health care provision, since ECT and NTIRE are highly successful modalities for treatment of malignant tumors and tissue ablation, respectively. The application presented here is portable and accessible, as it can be distributed either as a stand-alone software package that can be downloaded and installed, or as an online web application that is easily accessed from any computer possessing browsing capabilities. Feedback from students that have had an educational course of which ApiVizTEP is a part of, have provided feedback that confirms the usefulness and ease of use of the educational solution presented.

## Availability and requirements

**Project name:** ApiVizTEP

**Project home page:** e.g. http://sourceforge.net/projects/apiviztep/

**Operating system(s):** Web version: Platform independent, Desktop version: Microsoft Windows XP and above

**Programming language:** Web version: PHP (GUI) and C# (service), Desktop version: C#

**Other requirements:** Web version: Apache Server, MySQL, XHTML, CSS, CodeIgniter library, IIS 7 Web Server, .NET Framework v4, Desktop version: .NET Framework v4

**License:** GNU General Public License v3

**Any restrictions to use by non-academics:** Desktop version is subject to GNU GPL v3 license. Web-based version is closed to all public access, due to it being part of a wider educational framework that is not in public domain.

## Appendix

This appendix provides a detailed mathematical derivation for electric field strength of two long thin parallel cylindrical conductors. The basic theory of electric field supporting this derivation may be found in [40] or derived by means of almost any comprehensive treatment of electrostatic field theory that is found in literature, e.g. [53].

**Figure 9:**
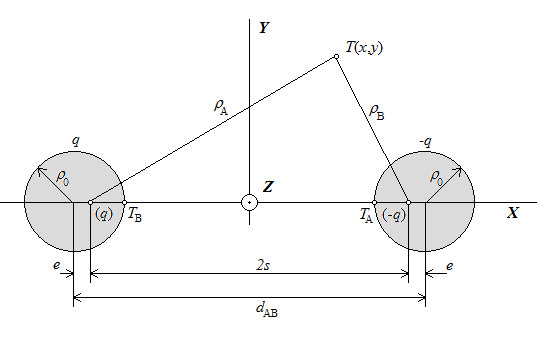
Two long parallel cylindrical electrical conductors.

The general situation is illustrated by Figure 9 below. We are looking for an analytical expression for the electric field of two long parallel cylindrical conductors of diameter *ρ*_0_ distance *d*_AB_ apart that carry electrical charges +*q* and –*q*. Charges +*q* and –*q* are the actual charges on the surface of the conductors, while charges (+*q*) and (−*q*) are their mathematical equivalents, which is why they are given in parentheses. These charges are located eccentrically to the geometrical axes of the conductors. The axes of the equivalent charges are also known as the *electrical axes* and the distance between these electrical axes and the geometrical axes is known as *eccentricity,* denoted *e.* Via means of the geometrical parameters of equipotential curves we determine the distance between the electrical axes, denoted by *s*, which we find is related to the existing system parameters as shown by the following equation:

$$s=\sqrt{{\left(\frac{d_{\mathrm{AB}}}{2}\right)}^{2}-{\rho_{0}}^{2}}\text{.}$$

From Figure 9 it also follows:

$$e=\frac{d_{\mathrm{AB}}}{2}-s=\frac{d_{\mathrm{AB}}}{2}-\sqrt{{\left(\frac{d_{\mathrm{AB}}}{2}\right)}^{2}-{\rho_{0}}^{2}}\text{.}$$

The electric potential in the area outside the conductors is defined by the equivalent charges:

$$V\left(T\right)=\frac{q}{2\pi \varepsilon_{0}}\log\frac{\rho_{B}}{\rho_{A}}\text{,}$$

and the voltage between the two conductors is the difference in potentials:

$$\begin{array}{l} V_{\mathrm{AB}}\left(T\right)=V\left(T_{A}\right)-V\left(T_{B}\right)=2\;V\left(T_{A}\right)\\ \;=\frac{q}{\pi \varepsilon_{0}}\log\frac{d_{\mathrm{AB}}+\sqrt{{d_{\mathrm{AB}}}^{2}-4{\rho_{0}}^{2}}}{2\rho_{0}}\text{.} \end{array}$$

If the voltage *V*_AB_(*T*) is known, the expression for the unknown potential *V*(*T*) is given by:

(1) $$V\left(T\right)=\frac{V_{\mathrm{AB}}\log\frac{\rho_{B}}{\rho_{A}}}{2\log\frac{d_{\mathrm{AB}}+\sqrt{{d_{\mathrm{AB}}}^{2}-4{\rho_{0}}^{2}}}{2\rho_{0}}}\text{.}$$

The electric field strength *E*(*T*) is the absolute value (length) of the vector ***E***(*T*), obtained by means of the gradient function of the scalar potential *V*(*T*):

(2) $$E\left(T\right)=\left|-grad\;V\left(T\right)\right|=\left|-\nabla V\left(T\right)\right|\text{.}$$

The gradient in two-dimensional space described using Cartesian coordinates *x* and *y* is:

(3) $$-grad\;V\left(x,y\right)=-\left(\frac{\delta V}{\delta x}+\frac{\delta V}{\delta y}\right)\text{.}$$

The gradient operation applies to the expression log(*ρ*_B_/*ρ*_A_) contained in A.1, where *ρ*_A_ and *ρ*_B_ are expressed as (see Figure 9):

(4) ρA=xA+e−x2+yA−y2=xA'−x2+yA−y2ρB=xB−e−x2+yB−y2=xB'−x2+yB−y2,

for the particular case where the *y* coordinates of the conductors’ geometrical axes are identical (and happen to be equal to 0, see Figure 9). If this is not the case – i.e. the coordinates of the geometrical centers are completely arbitrary – we need to determine the coordinates of the electrical axes via a rotational transformation (i.e. trigonometrically). For this general case, combining equations A.1 – A.4 and accounting for the rotational transformation to generalize eq. A.4, we get the following set of expressions that represent the final and general solution to our problem:

$$\left|E\left(x,y\right)\right|=C\cdot \sqrt{{\left(\frac{x{'}_{A}-x}{{\left(x{'}_{A}-x\right)}^{2}+{\left(y{'}_{A}-y\right)}^{2}}-\frac{x{'}_{B}-x}{{\left(x{'}_{B}-x\right)}^{2}+{\left(y{'}_{B}-y\right)}^{2}}\right)}^{2}+{\left(\frac{y{'}_{A}-y}{{\left(x{'}_{A}-x\right)}^{2}+{\left(y{'}_{A}-y\right)}^{2}}-\frac{y{'}_{B}-y}{{\left(x{'}_{B}-x\right)}^{2}+{\left(y{'}_{B}-y\right)}^{2}}\right)}^{2}}$$

$$\text{where}\;C=\frac{V_{\mathrm{AB}}}{2\cdot \log\frac{d_{\mathrm{AB}}+\sqrt{{d_{\mathrm{AB}}}^{2}-4\cdot {\rho_{0}}^{2}}}{2\cdot \rho_{0}}}$$

$$\begin{array}{ll} \text{and} & \begin{array}{ll} x{'}_{A}=x_{A}+\left|e\right|\cdot \cos\Theta_{1}, & \Theta_{1}=\arctan\frac{y_{B}-y_{A}}{x_{B}-x_{A}}\\ x{'}_{B}=x_{B}+\left|e\right|\cdot \cos\Theta_{2}, & \Theta_{2}=\arctan\frac{y_{A}-y_{B}}{x_{A}-x_{B}}\\ \begin{array}{l} y{'}_{A}=y_{A}+\left|e\right|\cdot \sin\Theta_{1},\\ y{'}_{B}=y_{B}+\left|e\right|\cdot \sin\Theta_{2} \end{array} & e=d_{\mathrm{AB}}/2-\sqrt{{\left(d_{\mathrm{AB}}/2\right)}^{2}-{\rho_{0}}^{2}} \end{array} \end{array}$$

In eq. A.5 – A.7, *V*_AB_ is the voltage on an electrode pair (anode–cathode), *d*_AB_ is the distance between the electrodes, *ρ*_0_ is the electrode diameter, while *x*’_A_, *y*’_A_ and *x*’_B_, *y*’_B_ are coordinates of the center of the electrode mathematical equivalents (the electrical center *x*’_A_, *y*’_A_ and *x*’_B_, *y*’_B_ of the electrodes is eccentric to the actual geometrical center *x*_A_, *y*_A_ and *x*_B_, *y*_B_ due to finite i.e. non-zero electrode diameter).

## Abbreviations

ECT: ElectroChemoTherapy; NTIRE: Non-Thermal IRreversible Electroporation; EBTT: Electroporation-Based Technologies and Treatments.

## Competing interests

The authors declare they have no competing interests.

## Authors’ contributions

SMK developed and tested the application and wrote the manuscript. TK contributed to the design of the application, tested the application and reviewed the manuscript. DM contributed to the design of the application, tested the application and reviewed the manuscript. All authors have read and approved the final manuscript.

## Pre-publication history

The pre-publication history for this paper can be accessed here:

http://www.biomedcentral.com/1472-6920/12/102/prepub
